# Case Report : Ongoing Delirium and Hypercalcaemia in a Patient With Bipolar Disorder Receiving Long Term Lithium Therapy

**DOI:** 10.1192/bjo.2026.11859

**Published:** 2026-06-30

**Authors:** Mohamed Fareeth, Hafijur Rahman, Fathima Zainab, Inaam Alam, Abdul Gafoor Mohamed Fareeth

**Affiliations:** 1 Trakia University, Stara Zagora, Bulgaria; 2 European University, Tiblisi, Georgia; 3 Plovdiv University, Plovdiv, Bulgaria; 4 King AbdulAziz, Al Ahsa, Saudi Arabia

## Abstract

**Aims:**

Lithium is a first-line mood stabiliser for bipolar disorder but has a narrow therapeutic index. Neuropsychiatric toxicity may occur even when serum lithium concentrations are near the therapeutic range, particularly in the presence of dehydration and acute kidney injury. This case study aims to highlight lithium-associated neuropsychiatric toxicity as an important and potentially overlooked cause of ongoing delirium in patients with bipolar disorder following acute medical illness.

**Methods:**

A 74-year-old woman with bipolar disorder treated with long-term lithium therapy was admitted following collapse and acute onset confusion. On presentation, she was febrile, dehydrated, and disoriented, with clinical evidence of cellulitis, leading to an initial diagnosis of acute delirium secondary to sepsis. She was treated empirically with intravenous antibiotics and fluids. Despite improved inflammatory markers, her neuropsychiatric symptoms persisted.

Further investigations revealed mild hypernatraemia, persistent hypercalcaemia, acute kidney injury, and a mildly elevated serum lithium level but her parathyroid hormone levels were within the normal limit. CT and MRI of the brain revealed no acute intracranial pathology. Despite rehydration and resolution of infection, delirium and biochemical abnormalities persisted, prompting review of psychotropic medication.

**Results:**

Lithium toxicity precipitated by dehydration and acute kidney injury was identified as the most likely unifying diagnosis. Lithium dosage was reduced and intravenous bisphosphonate therapy was administered. Following intervention, renal function improved and serum lithium and calcium concentrations normalised. The patient demonstrated gradual and complete resolution of delirium, returning to her cognitive baseline without residual neuropsychiatric impairment at discharge.

This case illustrates that lithium-associated neuropsychiatric toxicity may present with persistent delirium despite near-therapeutic serum levels and apparent alternative medical explanations. Hypercalcaemia and renal impairment served as important diagnostic clues supporting lithium toxicity.

**Conclusion:**

Lithium toxicity should be considered in patients with bipolar disorder who develop persistent delirium, particularly when dehydration and acute kidney injury are present. Near-therapeutic serum lithium levels do not exclude clinically significant neurotoxicity. Early review of psychotropic medication and renal function is essential to prevent delayed diagnosis and avoidable morbidity.

